# Follicle-Stimulating Hormone in Peripheral Metabolism: Novel Insights into Growth Regulation and Potential Applications in Boar Production

**DOI:** 10.3390/ani16071004

**Published:** 2026-03-25

**Authors:** Ganchuan Wang, Xingfa Han, De Wu, Yong Zhuo

**Affiliations:** 1 Animal Nutrition Institute, Sichuan Agricultural University, Chengdu 611130, China; 2023314118@stu.sicau.edu.cn (G.W.);; 2 School of Life Sciences, Sichuan Agricultural University, Yaan 625014, China

**Keywords:** surgical castration, immunocastration, FSH, GnRH, swine

## Abstract

Gonad loss triggers severe endocrine disorders and altered energy metabolism, yet the precise mechanisms remain poorly understood. In swine production, surgical castration is widely performed to eliminate boar taint and aggressive behavior, but it impairs feed efficiency, increases fat deposition, and raises animal welfare issues. Castration reduces testosterone and estrogen levels, leading to elevated gonadotropin-releasing hormone (GnRH) and its downstream follicle-stimulating hormone (FSH) and luteinizing hormone (LH). Traditionally viewed as a reproductive hormone, FSH has recently emerged as a critical regulator of peripheral metabolism. Based on these findings, we designed and developed a novel FSH vaccine comprising an FSHβ13AA-tandem-ovalbumin conjugate, which has been demonstrated to effectively regulate growth and metabolism in castrated boars. In conclusion, this review underscores the previously underrecognized metabolic functions of follicle-stimulating hormone (FSH) and proposes a novel immunomodulatory strategy targeting FSH for fine-tuning organ function and energy metabolism. This approach shows considerable potential for advancing sustainable, welfare-oriented swine production.

## Simple Summary

Male pigs are often surgically neutered to reduce unpleasant odors and behaviors, but this harms how they use feed, makes them gain too much fat, and raises animal welfare worries. The exact way in which removing testes/gonads affects body processes such as energy metabolism is not fully understood. This review focuses on follicle-stimulating hormone (FSH), a hormone initially recognized for promoting the development of female reproductive cells. New research shows it also affects how fat is processed, bone health, and liver function. Scientists made a new vaccine targeting this hormone using a small, shared part of it. Tests on neutered male pigs proved the vaccine could adjust their growth and body processes. This work reveals new roles of this hormone in body metabolism and offers a new vaccine-based method to improve pig farming, making it more sustainable by solving the problems from neutering, benefiting both animal welfare and the livestock industry.

## 1. Introduction

As the central reproductive and endocrine organs in mammals, gonads secrete sex hormones that regulate growth, metabolism, body composition, and behavior. They play an indispensable role in physiological health and livestock production. In the swine industry, however, surgical castration remains the most widely adopted practice, primarily aimed at mitigating boar taint in sexually mature boar meat and suppressing androgen-mediated behaviors (e.g., fighting and mounting [1,2,3]).

With the continuous advancement of livestock breeding technology, various technical methods have been developed to reduce or eliminate boar taint, but commercially available products remain relatively limited. The main technical approaches are briefly summarized as follows: (i) Sperm sorting technology: Based on the weight difference between X and Y sperm, flow cytometry is used for precise sperm sorting. Artificial insemination with only X sperm can produce all-female offspring, fundamentally avoiding the occurrence of boar taint [4] However, this technology has not yet achieved commercial popularization, with the core limiting factors being its long operation time and high cost [1]; (ii) Genetic selection technology: Androstenone and skatole, the two key substances responsible for boar taint, exhibit moderate to high heritability. Therefore, systematic breeding and cultivation of boars with low-taint phenotypes can be conducted to achieve genetic improvement of boar taint. Nevertheless, this method also suffers from the drawbacks of a long breeding cycle and high cost, and no commercial products have been developed to date [2]; (iii) Pre-pubertal slaughter: Slaughtering boars before they reach sexual maturity (at a body weight of approximately 100 kg) can effectively prevent the accumulation of boar taint-related substances; (iv) Surgical castration: Surgical castration of boars is a traditional classic method for controlling boar taint; (v) Immunocastration: Immunization induces the production of anti-gonadotropin-releasing hormone antibodies in boars, thereby inhibiting gonadal development and sex hormone secretion, and reducing the synthesis and release of taint substances such as androstenone and skatole. It serves as a novel alternative to surgical castration.

A report published by the European Commission in March 2019 indicated that although some EU Member States have gradually adopted entire male pig production systems or immunocastration technology, surgical castration remains the predominant practice in the swine industry across most Member States [4].

Surgical castration induces profound changes in the reproductive physiology of pigs, including alterations in the secretion patterns of GnRH and its downstream hormones FSH and LH, with elevated FSH being a characteristic feature [5,6]. While traditional immunocastration strategies primarily target the GnRH axis to regulate boar taint and growth performance, emerging evidence—including landmark studies in Nature [7]—has revealed that FSH acts as a critical metabolic regulator beyond its classical reproductive functions. In addition to regulating lipid metabolism, FSH also governs the development and function of multiple non-reproductive tissues, including liver, skeletal muscle, and bone [8]. Our recent work using a porcine FSH-specific vaccine further demonstrated that FSH may be a key factor contributing to the decreased feed conversion efficiency and increased lipid deposition observed in castrated boars [9].

Against this background, the present review provides a novel and timely perspective by focusing on FSH as a key metabolic regulator and promising vaccine target, which differs fundamentally from conventional GnRH-based immunocastration strategies. We systematically summarized the non-reproductive functions of FSH, clarified the differences in mechanisms between FSH immunocastration and traditional GnRH-based immunocastration, and discussed the application potential of targeting FSH in improving feed efficiency, increasing lean meat yield, and promoting the sustainable development of the global pig industry.

## 2. Surgical Castration

### The Influence of Surgical Castration on the Growth Performance, Carcass Traits and Meat Quality of Fattening Pigs

Pauly et al. [3] reported that during the growth phase of 21.6 to 105.8 kg (±0.9 kg), surgically castrated boars exhibited a 7.7% increase in overall average daily gain (ADG) compared with intact boars. However, significant elevations of 11.2%, 19.3%, and 70% were observed in overall feed conversion ratio (FCR), average daily feed intake (ADFI), and backfat thickness, respectively. Another study focusing on pigs weighing 30 to 120 kg demonstrated no significant differences in ADG or hot carcass weight between surgically castrated boars and intact boars, whereas the FCR of castrated males was 15.0% higher than that of intact boars [10]. Collectively, intact boars generally have an ADG that is lower than or comparable to that of surgically castrated boars, but they exhibit superior feed conversion efficiency, which is consistent with the findings of numerous studies [11,12,13,14]. Furthermore, surgically castrated boars excrete 15% more nitrogen than intact boars [15]. These results indicate that the feeding costs and environmental impacts associated with surgically castrated boars are significantly increased.

For finishing pigs, whole-body protein deposition is not only directly associated with lean growth efficiency and carcass economic value but also exerts a pivotal impact on the overall efficiency and profitability of pork production [16]. The protein deposition rate serves as the core determinant of amino acid nutritional requirements in pigs. As confirmed by the NRC (2012) [16] and numerous studies [17], the PDR of intact boars is higher than that of boars castrated shortly after birth.

In addition to affecting protein deposition, castration of boars increases carcass fat deposition and alters the characteristics of fatty acid composition. Systematic research has been conducted to elucidate the effects of surgical castration on porcine carcass traits and meat quality: a review published in 2009 comprehensively summarized the relevant research findings [15]; furthermore, Pauly et al. (2012) [18] performed a meta-analysis by integrating data from 28 published studies, providing a quantitative assessment of these effects. Collectively, compared with intact boars, surgically castrated boars exhibit a higher dressing percentage but have approximately 5% more separable carcass fat, which directly reduces their market value [3,13,19,20,21]. Additionally, the backfat of surgically castrated boars has lower protein content, higher lipid content, and a significantly decreased proportion of polyunsaturated fatty acids [3,13,19,20,21], rendering their dry-cured products less susceptible to rancidity during storage and maturation [18]. However, pork derived from surgically castrated pigs contains a relatively high concentration of saturated fatty acids, a property that may impose potential adverse impacts on human health [18,22]. From the perspective of meat quality improvement, surgically castrated boars have significantly higher intramuscular fat content than intact boars, and intramuscular fat content is a key positive factor influencing eating quality traits such as meat flavor and tenderness [18].

## 3. GnRH Immunocastration

### 3.1. The Mechanism of GnRH Immunocastration

Immunocastration via active immunization against GnRH is recognized as a potential strategy to ablate the mammalian reproductive system [23]. In 1986, investigators demonstrated that immunocastration using luteinizing hormone-releasing hormone (LHRH) conjugated to bovine serum albumin effectively prevented boar taint [24].

Immunocastration essentially leverages the animal’s innate immune system to achieve castration effects. A key component of immunocastration vaccines is a structurally modified, physiologically inert GnRH analog, which is covalently coupled to an immunogenic carrier protein to form an immunoconjugate [25]. Due to conformational alterations, such GnRH analogs are unable to bind to GnRH receptors on the surface of pituitary gonadotrophs and thus lack the hormonal activity of endogenous GnRH. Nevertheless, they retain the core antigenic epitopes of GnRH, enabling them to stimulate the immune system to produce high-titer, highly specific anti-GnRH antibodies. Upon specific binding of these antibodies to endogenous GnRH, inactive immune complexes are formed, thereby specifically blocking the signal transduction pathway of the hypothalamic-pituitary-gonadal (HPG) axis. This process directly inhibits the synthesis and secretion of steroid hormones (androgens, estrogens, or progestogens) in the gonads (testes or ovaries), subsequently inducing degenerative atrophy of reproductive organs and triggering a series of adaptive metabolic remodeling events. Ultimately, this results in significant improvements in animal behavioral traits (e.g., reduced aggressiveness, enhanced feeding motivation, and increased feed intake) as well as optimized growth performance [26].

To effectively block boar sexual development and reduce boar taint, immunological intervention with at least two doses of anti-gonadotropin-releasing hormone (anti-GnRH) vaccine is required. The primary dose is recommended to be administered at 8–10 weeks of age, mainly to initiate the immune response. The interval between the two doses should be no less than 4 weeks, and the booster dose (second dose) is typically given 4–6 weeks prior to slaughter. Studies have demonstrated that within several days following the booster vaccination, the behavioral characteristics of immunized boars become comparable to those of surgically castrated boars: aggressive and mounting behaviors are significantly reduced, while feed intake is markedly increased, with this feed intake level being higher than that of surgically castrated boars at the same growth stage [27,28,29,30].

### 3.2. Effects of GnRH Immunocastration on Growth Performance, Carcass Traits, and Meat Quality in Finishing Pigs

Based on research data spanning the entire finishing period (from the first immunization to slaughter), a meta-analysis investigating the effects of immunocastration on the growth performance of pigs revealed that the growth rate of immunocastrated pigs was statistically significantly higher than that of surgically castrated pigs and intact boars [31]. The underlying mechanism can be explained as follows: Prior to the administration of the second effective immunization, immunocastrated pigs maintain the physiological characteristics of intact males, thereby enabling the full exertion of the inherent growth potential of boars during this phase. Following the second immunization, significant modulation of reproductive hormone levels occurs, characterized by a rapid decline in steroid hormone concentrations [32]. Concurrently, the concentrations of insulin-like growth factor-1 (IGF-1) and growth hormone (GH) in pigs remain at relatively high levels [33,34].

This hormone regulatory profile results in a phenotypic difference in immunocastrated pigs—lower ADFI but higher ADG—compared to surgically castrated pigs after the full manifestation of the immunological effect, thereby achieving an optimized integration of growth performance. Consequently, the feed conversion efficiency of immunocastrated pigs is significantly superior to that of surgically castrated pigs. Studies by Batorek et al. [35] confirmed that successful immunocastration leads to a significant increase in adipose tissue deposition and a decrease in energy metabolism in animals. The protein deposition in immunocastrated pigs shows no significant difference from that in intact males but exhibits a statistical difference from surgically castrated animals, which tend to prioritize fat deposition over protein synthesis. However, it is crucial to note that the aforementioned findings are all based on the late immunocastration protocol, where the first immunization is initiated at the early stage of the finishing period and the second immunization is administered relatively late (typically 4–6 weeks before slaughter). This implies that early immunocastration may result in distinct physiological phenotypes. Currently, research on early immunocastration is relatively scarce, primarily due to its low economic feasibility [36].

The fat deposition characteristics of immunocastrated animals are correlated with the interval from the second immunization to slaughter. Existing studies have confirmed that a longer interval results in immunocastrated animals exhibiting phenotypic traits more similar to those of surgically castrated individuals, with a concomitant increase in fat deposition [37,38,39,40]. Although moderate intramuscular fat (IMF) deposition is recognized to improve meat sensory quality (e.g., tenderness, flavor), an elevation in overall body fat percentage exerts adverse effects on breeding economic efficiency—body fat percentage is negatively correlated with lean meat percentage, which serves as the core indicator determining carcass market value. A meta-analysis conducted by Batorek et al. [31] based on 30 relevant studies revealed that the backfat thickness of immunocastrated animals is significantly greater than that of intact males, directly leading to a reduction in carcass lean meat percentage. However, compared with surgically castrated animals, immunocastrated animals possess distinct advantages in carcass quality, specifically characterized by lower total carcass fat content and a higher weight proportion of high-value cuts such as ham and shoulder. Currently, the key technical approach for regulating fat deposition in immunocastrated animals involves dietary nutrient manipulation following the second immunization: both feed restriction [33] and reduction of dietary net energy content [41] can effectively mitigate adipose tissue deposition, thereby improving carcass lean meat percentage.

Meta-analysis results [31,42] have demonstrated that immunocastrated animals exhibit no significant differences from surgically castrated counterparts in key meat quality traits, including water-holding capacity, pH value, and meat color, showing a high degree of consistency. Compared with intact boars, immunocastration not only effectively eliminates boar taint—a core issue impairing meat palatability—but also significantly improves meat quality characteristics, specifically manifested by increased intramuscular fat content and enhanced meat tenderness. Importantly, immunocastrated animals exhibit a higher proportion of saturated fatty acids in adipose tissue, a fatty acid composition feature that holds important application advantages from the perspective of meat processing technology. Furthermore, compared with intact boars, immunocastrated animals can tolerate a higher slaughter age, and their meat products better meet the raw material requirements for dry-cured meat products in terms of quality stability and processing adaptability (such products have specific requirements for meat fat content, fatty acid composition, and processing tolerance). Previous studies [43,44,45,46] have further confirmed that the meat and fat quality (including fat content and fatty acid composition) of immunocastrated animals show no statistical differences from those of surgically castrated animals, and their meat products exhibit good stability during prolonged aging. Thus, immunocastrated animals are considered suitable raw materials for dry-cured meat product processing.

## 4. Why FSH Immunization Was Selected

### 4.1. New Insights into the Reproductive Hormone FSH

FSH, together with thyroid-stimulating hormone (TSH), luteinizing hormone (LH), and human chorionic gonadotropin (hCG), belongs to the glycoprotein hormone subfamily. Traditionally, FSH, LH, and hCG have been classified as reproduction-related hormones. Among these, the expression of follicle-stimulating hormone receptor (FSHR) was initially thought to be restricted exclusively to the gonads [47]. Consequently, FSH has long been regarded as one of the major regulatory factors governing gonadal function in both males and females [47].

From a molecular structure perspective, FSH is a heterodimeric glycoprotein composed of α and β subunits [48]. Significant heterogeneity exists in the glycosylation of FSH, with the major modification sites including asparagine 52 (Asn52) and asparagine 78 (Asn78) in the α subunit, as well as asparagine 7 (Asn7) and asparagine 24 (Asn24) in the β subunit [49,50]. In the same species, the α-subunit of FSH shares high homology with those of the other two glycoprotein hormones secreted by the pituitary gland, namely LH and TSH; in contrast, the β-subunit is specific and confers unique biological activity to each hormone [47]. The physiological role of FSH in reproductive processes was first validated by the following studies: FSH isolated from sheep was shown to increase the follicular volume of ovaries in hypophysectomized rats [51]; partially purified FSH derived from human pituitaries exhibited biological activity, as evidenced by its ability to elevate urinary estrogen levels in amenorrheic women, promote uterine cavity dilation, and induce polycystic enlargement of the ovaries [52]. These early studies and subsequent related experiments have laid a crucial foundation for clarifying the reproductive regulatory functions of FSH: within the female reproductive system, FSH acts as a key regulatory hormone for the maturation of Graafian follicles by promoting granulosa cell proliferation and the aromatization of androgens to estrogens; in the male reproductive system, FSH synergizes with testosterone to regulate spermatogenesis through modulating Sertoli cell function [53,54,55].

**Figure 1:**
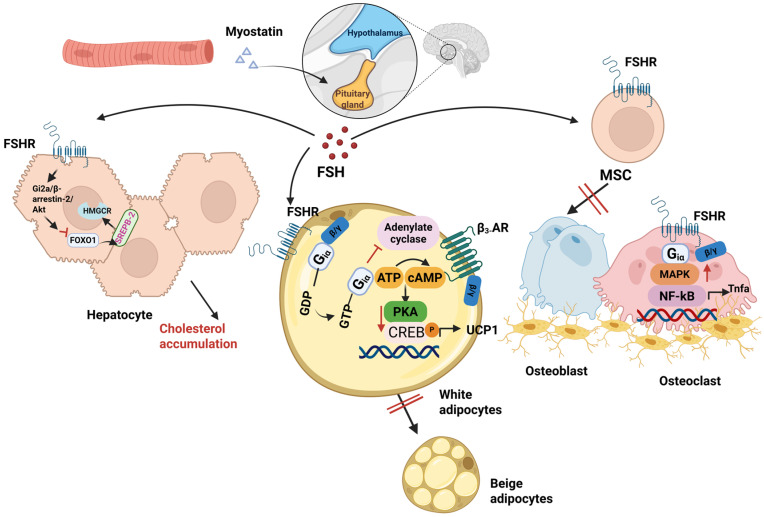
The effect of follicle-stimulating hormone (FSH) on bone, fat, and liver metabolism. This schematic was generated using BioRender (https://app.biorender.com/, accessed on 15 December 2025).

However, over the past two decades, the hypothesis regarding the functional specificity of FSH in reproductive processes has been increasingly challenged. With the continuous advancement of molecular biology techniques, methods such as immunohistochemistry, quantitative real-time polymerase chain reaction, Western blotting, and single-cell sequencing have been widely applied in the detection of receptor expression. A growing body of studies has confirmed the extragonadal expression of FSHR in tissues including the extragonadal reproductive tract, blood vessels, endothelial cells, liver, bone tissue, adipose tissue, and brain tissue [7,56,57,58,59]. FSH and FSHR are widely distributed across multiple species, such as mammals, birds, fish, reptiles, and amphibians; in certain tissues, FSHR can bind to both FSH and LH [60,61]. Additionally, these glycoprotein hormone receptors exhibit high structural homology among different species. For instance, the thyroid-stimulating hormone receptor (TSHR) of striped bass can be effectively activated by bovine thyroid-stimulating hormone [62]. Collectively, these findings have opened up broad research avenues for exploring the novel biological functions of glycoprotein hormones (Figure 1).

#### 4.1.1. FSHR and FSH in Adipose Tissue and Adipocytes: Regulation of Adipose Metabolism and Thermogenesis

As a crucial endocrine and energy-storage organ, adipose tissue plays a pivotal role in maintaining systemic energy balance and metabolic homeostasis [63,64]. Studies have demonstrated that FSHR is expressed in both white adipose tissue (WAT) and brown adipose tissue (BAT) [7,59]; however, the expression level of FSHR in adipocytes and adipose tissue is lower than that in the ovary [8].

FSHR expressed in adipose tissue and adipocytes can regulate adipometabolism and thermogenesis. Previous research has demonstrated the presence of FSHR transcripts and protein in abdominal adipose tissue of hens, whereas *FSH* gene expression was undetectable. Additionally, *FSH* was found to colocalize with FSHR in this tissue. Furthermore, chicken preadipocytes exhibit a linear response to changes in FSH concentration: when FSH concentration is doubled, the mRNA expression of FSHR, FSHR protein abundance, and lipid accumulation in preadipocytes increase linearly, accompanied by accelerated morphological differentiation into mature adipocytes [59]. Furthermore, in FSH-treated preadipocytes, the expression levels of lipid metabolism-related genes were significantly altered, including retinol dehydrogenase 10 (Rdh10) and dienoyl-CoA isomerase (Dci) involved in retinoic acid metabolism; retinoic acid receptor β (RARβ) and lipoprotein lipase (LPL) associated with the peroxisome proliferator-activated receptor signaling pathway; acyl-CoA synthetase long-chain family member 3 (Acsl3) implicated in fatty acid metabolism; and diacylglycerol acyltransferase 2 (DGAT2).

In particular, these genes play pivotal roles in key adipogenic processes such as fatty acid synthesis and lipid droplet formation [59]. In in vivo experiments, administration of chicken FSH to chicks resulted in a significant increase in abdominal fat weight, accompanied by a marked upregulation of FSHR transcripts in abdominal adipose tissue [59]. These findings confirm, from both in vivo and in vitro perspectives, that FSH-FSHR binding promotes lipid droplet formation in preadipocytes and the expression of adipogenesis-related genes. Furthermore, this process may be mediated through retinol metabolism, fatty acid metabolism, and the peroxisome proliferator-activated receptor (PPAR) signaling pathway.

In addition, FSH can induce lipid synthesis by upregulating the expression of adipogenic differentiation marker genes, including peroxisome proliferator-activated receptor γ (PPARγ), CCAAT enhancer-binding protein α (C/EBPα), LPL, fatty acid synthase (FAS), and perilipin [7]. Further studies have demonstrated that following binding to the FSHR, FSH exerts its biological effects through coupling with Gαi proteins. This interaction subsequently stimulates calcium ion influx, induces the phosphorylation of cyclic AMP response element-binding protein (CREB), and ultimately activates a cascade of genes involved in lipid biosynthesis [57]. Concurrently, in vitro experiments have observed that FSH promotes lipid droplet formation in 3T3-L1 preadipocytes in a concentration-dependent manner. Specifically, this pro-lipogenic effect is abrogated upon FSHR knockout, which further validates the specificity of this signaling pathway [57].

In addition to promoting adipogenesis, FSHR in adipocytes can also couple with Giα proteins to downregulate intracellular cyclic adenosine monophosphate (cAMP) levels [7], thereby inhibiting cAMP-mediated β3-adrenergic receptor signaling. Suppression of this signaling pathway results in decreased expression of uncoupling protein 1 (UCP1)—a key mitochondrial functional protein, which in turn inhibits the thermogenic capacity and the beigeing process of white adipocytes, ultimately leading to body fat accumulation [7].

In functional loss experiments, two intervention approaches were employed to attenuate the biological functions of FSH: (1) a gene-editing strategy using FSHR^+^/^−^ mice, and (2) a pharmacological intervention with FSH-specific blocking antibodies in mouse models fed a high-fat diet or subjected to ovariectomy. Consistent results were obtained from both approaches: these interventions upregulated UCP1 expression and promoted mitochondrial biogenesis, ultimately increasing energy expenditure and reducing fat deposition in preventive models [7]. Particularly, FSH blockade did not affect animal satiety; on the contrary, feed intake of the experimental mice showed an increasing trend, despite which a reduction in body weight was still achieved [7]. Collectively, these findings confirm that targeted blockade of FSH signaling in vivo may serve as a potential intervention strategy for reducing adipose tissue deposition.

#### 4.1.2. The Impact of FSH and FSHR in Hepatocytes on Hepatic Metabolism

The liver serves as the central organ for maintaining cholesterol homeostasis in the body, with its core functions encompassing key metabolic processes such as cholesterol biosynthesis, uptake, transformation, transport, and esterification [65]. Dysfunction of the liver is closely associated with the development of cardiovascular diseases, including hypercholesterolemia and atherosclerosis [8].

A 2018 study [58] demonstrated that ovariectomy—a murine model mimicking postmenopausal or hypoestrogenic states—induces systemic depletion of 17β-estradiol (E2) signaling accompanied by elevated FSH secretion. Remarkably, the hepatic effects of this hormonal perturbation are independent of hepatocyte-intrinsic estrogen receptors, as targeted deletion of estrogen receptors in hepatocytes fails to recapitulate the downstream phenotypes. Elevated FSH acts as a pivotal mediator of hepatic glucocorticoid (GC) hypersensitivity: administration of FSH alone to mice with intact ovarian function is sufficient to replicate the hepatic GC-hypersensitive phenotype observed in ovariectomized counterparts. Mechanistically, FSH enhances glucocorticoid receptor (GR)-mediated transcriptional activity by regulating both the chromatin recruitment efficiency and ligand-dependent phosphorylation status of GR, thereby markedly augmenting hepatic sensitivity to circulating GCs. Hyperactivated GR subsequently triggers hepatic lipid metabolic dysregulation, culminating in hepatic steatosis. Importantly, hepatocyte-specific GR knockout mice are completely resistant to ovariectomy-induced hepatic steatosis. Collectively, these findings establish that FSH mediates hepatic GR hypersensitivity in estrogen-deficient female mice, which in turn drives the development of hepatic steatosis.

As is well established, the biosynthesis of cholesterol initiates with acetyl-coenzyme A (acetyl-CoA), wherein 3-hydroxy-3-methylglutaryl coenzyme A reductase (HMGCR) serves as the key rate-limiting enzyme that regulates the flux of this synthetic pathway. Accumulating evidence has demonstrated that cAMP response CREB and sterol regulatory element-binding protein 2 (SREBP-2) are critical transcription factors involved in cholesterol metabolism. These two factors can specifically bind to the cyclic AMP response element and sterol regulatory element within the promoter region of the *HMGCR* gene, respectively [66,67]. As a major isoform of the SREBP family, SREBP-2 preferentially activates the expression of target genes involved in the cholesterol biosynthetic pathway, thereby modulating cholesterol synthesis [68].

Guo et al. [69] established a mouse model with elevated FSH levels, confirming that FSH can increase serum cholesterol independently of estrogen. Furthermore, blocking FSH signaling via anti-FSHβ antibodies or ablating the *FSHR* gene effectively prevented hypercholesterolemia induced by FSH injection or a high-cholesterol diet. Mechanistically, FSH binding to hepatic FSHR activates the Gi2α/β-arrestin-2/Akt signaling pathway, which in turn inhibits the binding of forkhead box O1 (FoxO1) to the sterol regulatory SREBP-2 promoter. This action relieves the suppressive effect of FoxO1 on SREBP-2 gene transcription, thereby upregulating SREBP-2 expression. As a key transcription factor, SREBP-2 drives the de novo transcription of HMGCR and de novo cholesterol synthesis, ultimately leading to increased cholesterol accumulation [69]. This study not only uncovers a novel function of FSH in hepatic cholesterol metabolism but also provides a new therapeutic strategy for hypercholesterolemia.

A 2023 study demonstrated that FSH not only binds to follicle-stimulating hormone receptors (FSHRs) on hepatocytes to modulate the de novo biosynthesis of hepatic cholesterol, but also acts in a local paracrine manner on pituitary corticotropes to suppress the synthesis and secretion of corticosterone, thereby indirectly regulating hepatic lipid metabolism [70]. Unraveling this mechanism provides a novel perspective for elucidating the role of FSH in hepatic steatosis and, concomitantly, offers an innovative therapeutic paradigm for fatty liver disease [70].

#### 4.1.3. FSHR in Osteoclasts and the Regulation of Bone Mass by FSH

The skeletal system is a critical tissue that maintains body morphology, protects internal organs, and participates in calcium-phosphorus metabolism. Bone mass homeostasis relies on the dynamic balance between bone formation by osteoblasts and bone resorption by osteoclasts [71,72]. A sophisticated regulatory network exists between tropic hormones secreted by the pituitary gland (e.g., TSH, ACTH, FSH) and their corresponding effector hormones produced by target glands (triiodothyronine [T3]/tetraiodothyronine [T4], cortisol, and estradiol, respectively). This poses a significant challenge to the specific elucidation of the direct biological effects of tropic hormones. Taking skeletal metabolism regulation as an example, both thyroid hormones (T3/T4) and estrogen have been well-documented to exert potent and pivotal regulatory effects on bone modeling and bone remodeling processes. This complexity renders the independent characterization of the direct roles of TSH or FSH in skeletal physiology particularly intricate.

Sun et al. [73] validated through experiments using FSHβ subunit haploinsufficient (Fshb+/–) mice that FSHR mRNA and protein are expressed in mouse osteoclasts and their precursor cells. Furthermore, in mice with intact uterine and ovarian functions and normal estradiol levels, circulating FSH reduction significantly increased bone mass, directly confirming the functional relevance of FSHR on osteoclasts and their precursors. Subsequent studies employing pharmacological interventions—administration of FSH to rodents exacerbated ovariectomy-induced bone loss, while injection of FSH antagonists alleviated this bone loss phenotype [74,75]—further demonstrated that FSH itself can directly modulate the extent of bone loss even under estrogen-deficient conditions. These findings indicate that the effect of FSH on bone is independent of estrogen mediation and instead exerts a direct action on bone cells.

Unlike ovarian granulosa cells, FSHR expressed in CD11b^+^ osteoclast precursor cells and mature osteoclasts couples with the inhibitory Gαi protein. Consequently, in *Gαi* gene knockout (Gαi^−^/^−^) cells, FSH-induced regulation of osteoclastogenesis and activation of the downstream mitogen-activated protein (MAP) kinase signaling pathway are both abrogated [73]. Activation of FSHR in osteoclasts enhances the nuclear localization of c-Fos protein and induces the phosphorylation of extracellular signal-regulated kinase 1/2 (Erk1/2) and protein kinase B (Akt) [73].

The osteoclastogenic effect of follicle-stimulating hormone (FSH) is also mediated, at least in part, by cytokines. Strikingly, FSH upregulates the expression of receptor activator of nuclear factor-κB (RANK) [76], while concomitantly promoting the production of tumor necrosis factor (TNF)-α, interleukin (IL)-1β and interleukin (IL)-6 [77,78]. Furthermore, in murine models deficient in the signaling pathway of immunoreceptor tyrosine-based activation motif (ITAM)-adaptor molecules, FSH fails to induce osteoclast differentiation [79]—a finding indicating that immunoreceptors play a critical role in the regulatory actions of FSH.

Unlike osteoclasts, the direct mechanism underlying the effect of FSH on osteoblasts remains controversial. In vitro cell experiments have confirmed that the FSHR is primarily expressed on the surface of osteoclasts, osteoclast precursors, and mesenchymal stem cells, whereas its expression is not detected in mature osteoblasts [80]. Functional studies further demonstrated that FSH significantly promotes the differentiation and maturation of osteoclasts, enhances their biological activity, and improves cell survival capacity, but exerts no significant regulatory effect on the physiological functions of mature osteoblasts [80]. However, in contrast to the findings in mature osteoblasts, FSHR expression has been detected in osteoblast precursors in some studies [8], and functional validation showed that FSH can inhibit the differentiation of osteoblast precursors into osteoblasts [75]. In both sham-operated and ovariectomized mice, treatment with FSH-blocking antibodies significantly increased key bone formation-related parameters, including mineralizing surface per bone surface, mineral apposition rate and bone formation rate [75].

Based on the comprehensive evidence from existing in vivo and in vitro experiments, FSH not only directly or indirectly promotes the differentiation and activity of osteoclasts by regulating inflammatory pathways but also inhibits osteoblast differentiation through acting on FSHR expressed on osteoblast precursor cells, ultimately participating in the regulation of bone metabolic homeostasis.

#### 4.1.4. The Association Between Muscle Tissue and FSH Synthesis

Myostatin, a member of the transforming growth factor-β (TGF-β) superfamily primarily secreted by skeletal muscle, has traditionally been recognized as a key negative regulator of muscle growth and development [81]. However, recent studies have revealed that myostatin functions not only as a paracrine factor modulating muscle mass but also as an endocrine hormone that directly promotes FSH synthesis in the pituitary gland [82]. While FSH synthesis was previously thought to be mainly regulated by activins, another subgroup of the TGF-β family, this study demonstrated that myostatin is the direct driver of this process, challenging the “exclusive role” of activins. Remarkably, it is the first study to explicitly identify that skeletal muscle regulates the upstream of the reproductive system (i.e., pituitary FSH synthesis) via endocrine signaling, thereby establishing a novel muscle-pituitary endocrine axis and opening new avenues for cross-system regulatory research. This finding also breaks the traditional paradigm that muscle solely mediates locomotion and metabolism, providing compelling evidence that muscle can regulate upstream reproductive functions through hormone secretion. Furthermore, this discovery suggests that myostatin may serve as a novel therapeutic target for reproductive disorders characterized by aberrant FSH synthesis, such as polycystic ovary syndrome and premature ovarian insufficiency, offering innovative strategies for the clinical management of these conditions.

### 4.2. Research Progress of FSH in Pigs

Currently, carcass fat deposition induced by surgical castration in pigs is generally attributed to the deficiency of testicular steroids [18,48]. Multiple reports have indicated that castration of piglets is typically performed within a few days after birth, resulting in a permanent elevation of FSH concentrations in the bloodstream [49,83]. Mechanistically, the failure of testosterone synthesis and secretion following surgical castration disrupts the testosterone-Kisspeptin-GPR54-GnRH signaling pathway, leading to the sustained inhibition of hypothalamic GnRH synthesis. Concurrently, the expression of gonadotropin-inhibitory hormone (GnIH) is downregulated, attenuating its inhibitory effect on the pituitary gland and thereby causing increased concentrations of LH and FSH [6].

Tissue expression profiling via reverse transcription-quantitative polymerase chain reaction (RT-qPCR) has confirmed the moderate expression of FSHR in both subcutaneous and visceral adipose tissues of pigs [84]. Notably, surgical castration not only induces a significant elevation in serum FSH concentrations but also triggers a sharp increase in LH levels in pigs. However, no LHR expression signal was detected in either subcutaneous or visceral adipose tissues by qPCR [85]. This finding was further validated using Super deepSAGE high-throughput sequencing technology, which demonstrated FSHR expression but absence of LHR expression in porcine subcutaneous/visceral adipose tissues [85]. These lines of evidence indicate that FSH may regulate adipose deposition in pigs through its receptor-mediated signaling pathway, whereas LH does not participate in this regulatory mechanism due to the lack of functional LHR in adipose tissues. Additionally, in a castrated mouse model treated with a GnRH agonist, exogenous recombinant FSH administration significantly increased body weight and fat mass [58]. This cross-species research evidence further supports the biological function of FSH, rather than LH, in promoting adipose deposition under castration conditions.

To further validate the association between elevated FSH concentrations and carcass fat deposition in surgically castrated animals, this study investigated the effects of surgical castration (characterized by high FSH levels) versus immunocastration (characterized by low FSH levels) on adipose accumulation in the carcass. Consistent with our hypothesis, surgical castrates exhibited significantly higher carcass fat deposition compared to immunocastrates, which was paralleled by marked differences in serum FSH concentrations between the two groups. This finding aligns with the conclusions reported in the majority of previous studies [5,19,25,31,86]. Additionally, serum concentrations of triglycerides (TGs) and high-density lipoprotein (HDL) were significantly elevated in surgically castrated animals relative to immunocastrated counterparts. Collectively, these results support the preliminary speculation that the sharp increase in endogenous FSH concentrations following surgical castration may serve as one of the key regulatory factors contributing to enhanced carcass fat deposition. This conjecture is corroborated by comparable research findings: studies have demonstrated that male animals subjected to surgical castration exhibit higher carcass fat content than those immunized against GnRH, a treatment paradigm that is inherently associated with a significant reduction in serum FSH concentrations [87]. Therefore, the substantial increase in fat deposition observed in boars after surgical castration is at least partially attributable to the marked elevation of FSH levels. Conversely, the lower carcass fat deposition in immunocastrated boars compared to traditionally castrated counterparts is also at least partially associated with the sustained low serum FSH concentrations induced by immunocastration.

Liu et al. [57] utilized the 3T3-L1 preadipocyte in vitro model and demonstrated that following the binding of FSH to its receptor, PPARγ is activated via the Gαi-Ca^2+^-cAMP response CREB signaling pathway. This activation subsequently recruits a cascade of PPARγ downstream adipogenic genes, including C/EBPα, FAS, acetyl-CoA carboxylase α (ACACA), LPL, and perilipin 1 (PLIN1), ultimately promoting adipogenesis and lipid storage [57,88]. Based on the aforementioned research foundation, we further examined the mRNA expression profiles of the aforementioned key adipogenic genes in adipose tissues of boars following surgical castration and immunocastration [84].

**Figure 2:**
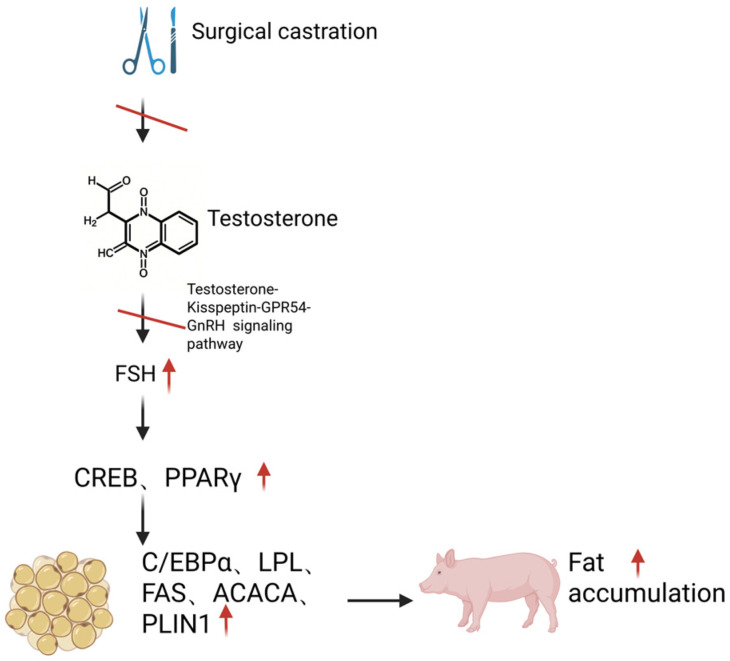
Schematic diagram illustrating how surgical castration modulates follicle-stimulating hormone (FSH) levels and thereby promotes fat deposition in boars. This schematic was generated using BioRender (https://app.biorender.com/, accessed on 15 December 2025). The upward-pointing arrow in the picture indicates an increase.

Our findings revealed that the drastic elevation of FSH concentration post-surgical castration significantly upregulated the mRNA expression of CREB and PPARγ, which in turn activated a cascade of PPARγ target genes in both subcutaneous and visceral adipose tissues, including C/EBPα, LPL, FAS, ACACA, and PLIN1. In contrast, immunocastration led to a marked reduction in FSH concentration, accompanied by decreased CREB mRNA expression; noteworthy, no significant difference was observed in PPARγ mRNA levels in subcutaneous or visceral adipose tissues between immunocastrated and intact boars. Consequently, compared with surgically castrated boars, immunocastrated boars exhibited substantially lower mRNA expression of PPARγ-targeted adipogenic genes (including C/EBPα, FASN, ACACA, LPL, and PLIN1), particularly in visceral adipose tissue. The differential expression of C/EBPα, PPARγ, LPL, and FASN among groups was further validated at the protein level via ELISA, with results consistent with those obtained from mRNA analysis. Collectively, these data lead us to conclude that FSH plays a pivotal regulatory role in adipogenesis and fat deposition in boars by activating the PPARγ signaling pathway [84] (Figure 2); furthermore, the difference in peripheral FSH concentrations between surgically castrated and immunocastrated boars is closely associated with their distinct carcass fat deposition capacities [84].

In addition to promoting adipogenesis, the reduced efficiency of lipolysis induced by castration may also be a crucial contributor to the abnormal carcass fat deposition. However, our results demonstrated that neither surgical castration nor immunocastration exerted a significant effect on the lipolytic process in pigs—this conclusion is supported by the expression profiles of key lipolysis-related genes in subcutaneous and visceral adipose tissues, specifically manifested by non-statistically significant minor fluctuations in the transcriptional levels of adipose triglyceride lipase (ATGL) and hormone-sensitive lipase (HSL) [84]. This finding is highly consistent with the results of a previous study on Yorkshire × Landrace crossbred pigs [89]. That study confirmed that surgical castration significantly upregulates the expression of key lipogenic genes, such as fatty acid synthase (FASN) and acetyl-CoA carboxylase α (ACCα), thereby enhancing de novo fatty acid synthesis. However, no significant differences in the expression levels of core lipolytic genes (including *HSL* and *ATGL*) were detected between castrated and intact control groups at 147 and 210 days of age [89]. Based on the aforementioned research evidence, we hypothesize that FSH is unlikely to mediate post-castration fat deposition by inhibiting lipolytic pathways.

### 4.3. Design, Preparation, and Practical Application of a Novel FSH Vaccine

As classic key hormones of the reproductive axis, GnRH and FSH have traditionally been focused on the development and application of immunocastration technologies. However, multiple independent studies in recent years have clearly demonstrated the previously unrecognized functions of FSH, including its significant role in promoting adipogenesis [7,57,58,59]. This groundbreaking discovery not only expands the physiological function spectrum of FSH but also provides a potential direction for targeting FSH to regulate fat deposition and treat obesity [8]. Combined with our previous research [84], we hypothesize that the compensatory elevation of endogenous FSH levels may induce reprogramming of systemic energy metabolism through its mediating effect on promoting adipogenesis. This metabolic shift subsequently leads to limited growth potential and reduced meat production performance, which might be one of the crucial contributing factors to the suboptimal productive performance of surgically castrated boars. Based on this premise, we aim to develop a novel FSH vaccine designed to block the regulatory function of FSH in adipogenesis, thereby improving the growth performance and meat production efficiency of castrated boars. This study not only provides an innovative technical solution for enhancing the economic benefits of pig production but also breaks through the traditional application limitations of FSH in the field of reproductive regulation, paving a new avenue for its research and application in metabolic regulation.

Grasso et al. (1998) employed isotope tracing methodology to identify two key structural domains on the human FSHβ subunit that mediate binding to the FSHR, corresponding to the amino acid sequences at positions 33–53 and 81–95 [90]. In a subsequent study, Liu et al. (2017) [7] further refined the FSHR-binding site on the FSHβ subunit to a 13-amino acid sequence through the construction of molecular computational models of the mouse and human FSHR-FSH complexes. To validate the functional significance of this sequence, the researchers generated polyclonal antibodies against this 13-amino acid peptide. In vitro experiments demonstrated that these antibodies could effectively block the specific binding between FSH and FSHR, directly confirming the central role of this sequence in the receptor-binding process [7]. FSHβ peptide sequences of major mammalian species were retrieved from the NCBI database. Conservation analysis performed using DNAMAN (Version 9) revealed that a 13-amino acid sequence is highly conserved across species [91]. Conspicuously, the key amino acid residues implicated in FSHR interaction, namely valine, tyrosine, aspartic acid, alanine, arginine, and lysine at the C-terminal position—previously identified in humans and mice [92]—exhibited 100% conservation among all analyzed species. These findings collectively demonstrate that this 13-amino acid sequence (hereinafter referred to as FSHβ13AA) constitutes a conserved core epitope within the FSHβ subunit that mediates FSHR binding across mammalian species.

Based on the aforementioned findings, we propose that compared with the native FSH protein, antigens or vaccines designed with FSHβ13AA as the core are expected to retain receptor-binding inhibitory activity while significantly reducing the non-specific immune responses potentially induced by the native protein. This provides a novel strategy for the development of innovative FSH-targeted immunological agents. Theoretically, vaccines or antibodies developed based on this conserved sequence may achieve effective immunoneutralization of endogenous FSH activity across different species. Therefore, we ultimately selected the FSHβ13AA sequence with the highest cross-species conservation as the hapten for the construction of novel FSH antigens and vaccines. Prominently, this cross-species conserved sequence is identical to the corresponding sequences of the FSHβ subunit in pigs and sheep, suggesting that immunological agents prepared using this sequence may have inherent compatibility with pigs and sheep, while also holding potential application value for other analyzed species, including humans. The processes of peptide synthesis, conjugation, and complete vaccine preparation were performed in accordance with the standardized experimental protocols established in our previously published study [91]. In summary, we designed a novel FSH antigen by concatenating the FSHβ13AA peptide (amino acid sequence: LVYKDPARPNIQK) and conjugating it with ovalbumin, designated as FSHβ13AA-T-OVA. This study lays a foundation for subsequent immunogenicity evaluation and functional validation experiments.

First, we evaluated the efficacy of the novel FSH vaccine in inhibiting fat deposition using ovariectomized (OVX) and normal mouse models. Our results demonstrated that immunization with the novel antigen emulsified in the mild adjuvant Specol effectively prevented body weight gain and fat accumulation in OVX mice (*p* < 0.01). Additionally, administration of this FSH vaccine significantly reduced fat deposition in normal male and female mice (*p* < 0.05). Mechanistically, two molecular pathways have been reported to mediate the pro-adipogenic effects of FSH. First, upon binding to its receptor on adipocytes, FSH upregulates cyclic AMP response CREB, which in turn activates PPARγ. This activation subsequently recruits a cascade of adipogenic genes, such as *FASN*, *ACACA*, *LPL*, and *Perilipin*, thereby directly promoting lipid biosynthesis [57,58]. Second, FSH suppresses the browning of white adipocytes and thermogenesis by inhibiting the expression of UCP1 [7]. Consistent with the aforementioned findings, our validation via quantitative real-time polymerase chain reaction (qPCR) demonstrated that elevated FSH levels in OVX mice activate PPARγ and recruit its target adipogenic genes in both visceral and subcutaneous adipose tissues, while suppressing UCP1 expression, ultimately contributing to the development of an obese phenotype. Vaccination with the FSH vaccine completely or significantly reversed FSH-induced PPARγ activation and UCP1 downregulation in OVX mice, thereby effectively preventing ovariectomy-induced obesity. Feed intake is a critical factor influencing animal body weight changes and fat deposition. In particular, FSH vaccination had no significant effects on the cumulative feed intake or fasted feed intake of OVX mice. This indicates that the reduced body weight and fat accumulation observed in vaccinated OVX mice are entirely attributed to the specific inactivation of endogenous FSH bioactivity, rather than modulation of feeding behavior. Mechanistically, the efficacy of the FSH vaccine relies on the induction of specific antibodies that exhibit high specificity for the β-subunit of FSH. These antibodies block the interaction between FSH and its receptors in target organs, as well as the activation of downstream signaling pathways, ultimately exerting anti-obesity effects. It is worth emphasizing that there was no significant alteration in the circulating FSH levels in the serum of immunized mice [7], which further confirms that the FSH vaccine operates through a “functional blocking” mechanism rather than “level downregulation”.

However, due to the species-specific differences in the immunogenicity and biological effects of adjuvants, the response patterns of different species to the same adjuvant may exhibit significant divergence. Therefore, direct validation in target animal models is required to clarify its actual efficacy. Based on this, we systematically evaluated the regulatory effects of a self-developed novel FSH vaccine on growth performance and meat quality-related traits in surgically castrated boars, a model characterized by significantly elevated endogenous FSH levels. The results showed that although vaccination with this vaccine exerted a certain impact on carcass meat quality, it could significantly improve the feed conversion efficiency of castrated boars at specific stages and effectively reduce body fat deposition [9]. Additionally, both the liver weight and liver index of boars in the FSH vaccine group exhibited statistically significant increases. This phenotypic change suggests that blocking the FSH signaling pathway may induce metabolic reprogramming associated with glycolipid metabolism or protein synthesis [9]. These findings provide experimental evidence for the FSH vaccine as a potential technical tool to regulate pig production efficiency and body composition, confirming its application feasibility in swine production.

Nevertheless, several critical translational hurdles must be overcome for the practical implementation of FSH immunization strategies. Foremost, safety remains a paramount consideration, necessitating further investigations to delineate potential reactogenicity, off-target effects, and long-term toxicity profiles. Additionally, the durability of immune responses warrants systematic evaluation, with longitudinal monitoring of humoral and cellular immunity being essential to ascertain the persistence of protective immunity and the requirement for booster regimens. Regulatory feasibility must also be rigorously assessed, encompassing compliance with current Good Manufacturing Practices (cGMP), scalable production, and alignment with established vaccine regulatory guidelines.

To improve industrial applicability, future studies should clarify how FSH immunization could be integrated into existing pig production systems, particularly in comparison with current GnRH-based immunocastration protocols. It is critical to determine whether FSH immunization serves as a supplementary strategy to conventional surgical castration or a potential alternative to castration. Additionally, the associated cost-effectiveness ratio and economic feasibility under commercial conditions should be evaluated.

Notably, a representative survey of Italian consumers revealed that while 54.5% expressed favorable attitudes toward immunocastration and 18.7% were willing to pay a premium for pork from immunocastrated pigs, 34.2% still perceived the technology as risky [93]. Accordingly, FSH immunization strategies will similarly encounter substantial challenges concerning public and consumer acceptance, which must be addressed through targeted communication and transparency regarding the mechanism and safety.

## 5. Conclusions

Surgical castration is widely applied in swine production to control boar taint and aggressive behavior, yet it causes severe endocrine and metabolic disorders, reduces feed efficiency, increases fat deposition, and raises animal welfare issues. Although various alternatives to surgical castration have been proposed, none have emerged as a globally optimal solution, and their application remains context-dependent on production systems, market demands, and social constraints.

This review highlights the non-canonical metabolic functions of FSH beyond reproduction, including its regulatory roles in adipose, hepatic, and bone metabolism. Our team has developed a novel FSH vaccine targeting a conserved epitope of the FSH β-subunit, aiming to ameliorate metabolic deficits and improve growth performance in castrated boars via immunomodulation.

Current evaluations of growing-finishing pigs focus on growth traits and meat quality, which collectively determine production efficiency and economic benefits. However, systematic studies on FSH immunomodulation regarding production performance and meat quality in surgically castrated pigs are still lacking, forming a major bottleneck for industrial translation.

Preliminary evidence from our group demonstrates that FSH immunization regulates meat quality-related traits, providing new strategies for improving both growth efficiency and meat quality. Collectively, this review supports FSH as a promising target for alleviating metabolic disturbances caused by castration. FSH-targeted immunomodulation represents an innovative, sustainable, and welfare-friendly approach to optimizing swine production, with great potential for practical application in the pig industry.

## Data Availability

No new data were created or analyzed in this study. Data sharing is not applicable.

## Abbreviations

The following abbreviations are used in this manuscript:

GnRH	Gonadotropin-releasing hormone
FSH	Follicle-stimulating hormone
ADG	Average daily gain
FCR	Feed conversion ration
ADFI	Average daily feed intake
TSH	Thyroid-stimulating hormone
LH	Luteinizing hormone
hCG	human chorionic gonadotropin
FSHR	Follicle-stimulating hormone receptor
TSHR	Thyroid-stimulating hormone receptor
WAT	White adipose tissue
BAT	Brown adipose tissue
Rdh 10	Retinol dehydrogenase 10
Dci	Dienoyl-CoA isomerase
RAR β	Retinoic acid receptor β
LPL	Lipoprotein lipase
Acsl 3	Acyl-CoA synthetase long-chain family member 3
DGAT 2	Diacylglycerol acyltransferase 2
PPAR	Peroxisome proliferator-activated receptor
PPAR γ	Peroxisome proliferator-activated receptor γ
C/EBPα	CCAAT enhancer-binding protein α
FAS	Fatty acid synthase
CREB	Cyclic AMP response element-binding protein
cAMP	cyclic adenosine monophosphate
UCP 1	Uncoupling protein 1
acetyl-CoA	acetyl-coenzyme A
HMGCR	3-hydroxy-3-methylglutaryl coenzyme A reductase
SREBP-2	Sterol regulatory element-binding protein 2
FoxO1	Forkhead box O1
MAP	Mitogen-activated protein
Erk1/2	Extracellular signal-regulated kinase 1/2
Akt	Protein kinase B
TGF-β	Transforming growth factor-β
GnIH	Gonadotropin-inhibitory hormone
TG	Triglycerides
HDL	High-density lipoprotein
ACACA	Acetyl-CoA carboxylase α
PLIN1	Perilipin 1
ATGL	Adipose triglyceride lipase
HSL	Hormone-sensitive lipase
FASN	Fatty acid synthase
ACCα	Acetyl-CoA carboxylase α
OVX	Ovariectomized

## References

[1] von Borell E., Baumgartner J., Giersing M., Jäggin N., Prunier A., Tuyttens F.A., Edwards S.A. (2009). Animal welfare implications of surgical castration and its alternatives in pigs. Animal.

[2] Duarte D.A.S., Schroyen M., Mota R.R., Vanderick S., Gengler N. (2021). Recent genetic advances on boar taint reduction as an alternative to castration: A review. J. Appl. Genet..

[3] Pauly C., Spring P., O’Doherty J.V., Ampuero Kragten S., Bee G. (2008). Performances, meat quality and boar taint of castrates and entire male pigs fed a standard and a raw potato starch-enriched diet. Animal.

[4] del Olmo D., Parrilla I., Gil M.A., Maside C., Tarantini T., Angel M.A., Roca J., Martinez E.A., Vazquez J.M. (2013). Handling of boar spermatozoa during and after flow cytometric sex-sorting process to improve their in vitro fertilizing ability. Theriogenology.

[5] Zeng X.Y., Turkstra J.A., Jongbloed A.W., van Diepen J.T.M., Meloen R.H., Oonk H.B., Guo D.Z., van de Wiel D.F.M. (2002). Performance and hormone levels of immunocastrated, surgically castrated and intact male pigs fed ad libitum high- and low-energy diets. Livest. Prod. Sci..

[6] Han X., Zhou Y., Zeng Y., Sui F., Liu Y., Tan Y., Cao X., Du X., Meng F., Zeng X. (2017). Effects of active immunization against GnRH versus surgical castration on hypothalamic-pituitary function in boars. Theriogenology.

[7] Liu P., Ji Y., Yuen T., Rendina-Ruedy E., DeMambro V.E., Dhawan S., Abu-Amer W., Izadmehr S., Zhou B., Shin A.C. (2017). Blocking FSH induces thermogenic adipose tissue and reduces body fat. Nature.

[8] Korkmaz F., Gimenez-Roig J., Sultana F., Laurencin V., Sen F., Cullen L., Sims S., Pallapati A., Rojekar S., Burganova G. (2025). Targeting FSH for osteoporosis, obesity, and Alzheimer’s disease. Trends Mol. Med..

[9] Wang G., Zhou J., Lv G., Jiang X., Song C., Hua L., Wang C., Jin C., Wu D., Han X. (2025). The Effects of FSH Versus GnRH Vaccination on Growth Performance and Meat Quality of Surgically Castrated Male Growing-Finishing Pigs. Animals.

[10] Zoels S., Reiter S., Ritzmann M., Weiß C., Numberger J., Schütz A., Lindner P., Stefanski V., Weiler U. (2020). Influences of Immunocastration on Endocrine Parameters, Growth Performance and Carcass Quality, as Well as on Boar Taint and Penile Injuries. Animals.

[11] D’Souza D.N., Mullan B.P. (2002). The effect of genotype, sex and management strategy on the eating quality of pork. Meat Sci..

[12] Dunshea F.R., Colantoni C., Howard K., McCauley I., Jackson P., Long K.A., Lopaticki S., Nugent E.A., Simons J.A., Walker J. (2001). Vaccination of boars with a GnRH vaccine (Improvac) eliminates boar taint and increases growth performance. J. Anim. Sci..

[13] Pauly C., Spring P., O’Doherty J.V., Ampuero Kragten S., Bee G. (2009). Growth performance, carcass characteristics and meat quality of group-penned surgically castrated, immunocastrated (Improvac^®^) and entire male pigs and individually penned entire male pigs. Animal.

[14] Bonneau M., Weiler U. (2019). Pros and Cons of Alternatives to Piglet Castration: Welfare, Boar Taint, and Other Meat Quality Traits. Animals.

[15] Lundström K., Matthews K.R., Haugen J.E. (2009). Pig meat quality from entire males. Animal.

[16] National Research Council (2012). Nutrient Requirements of Swine.

[17] Martínez-Ramírez H.R., Jeaurond E.A., de Lange C.F. (2008). Dynamics of body protein deposition and changes in body composition after sudden changes in amino acid intake: I. Barrows. J. Anim. Sci..

[18] Pauly C., Luginbühl W., Ampuero S., Bee G. (2012). Expected effects on carcass and pork quality when surgical castration is omitted—Results of a meta-analysis study. Meat Sci..

[19] Yao Y., Ma H., Wu K., Shao Y., Han W., Cai Z., Xu N., Qi M., Zhao C., Wu C. (2018). Body composition, serum lipid levels, and transcriptomic characterization in the adipose tissue of male pigs in response to sex hormone deficiency. Gene.

[20] Huber L., Squires E.J., Mandell I.B., de Lange C.F.M. (2018). Age at castration (surgical or immunological) impacts carcass characteristics and meat quality of male pigs. Animal.

[21] Babol J., Squires E.J. (1995). Quality of meat from entire male pigs. Food Res. Int..

[22] Poulsen Nautrup B., Van Vlaenderen I., Aldaz A., Mah C.K. (2018). The effect of immunization against gonadotropin-releasing factor on growth performance, carcass characteristics and boar taint relevant to pig producers and the pork packing industry: A meta-analysis. Res. Vet. Sci..

[23] Thompson D.L. (2000). Immunization against GnRH in male species (comparative aspects). Anim. Reprod. Sci..

[24] Caraty A., Bonneau M. (1986). Active immunization of male pigs against gonadoliberin: Effect on the secretion of gonadotropic hormones and on 5 alpha-androst-16-ene-one levels in adipose tissue. C. R. Acad. Sci. III.

[25] Gispert M., Angels Oliver M., Velarde A., Suarez P., Pérez J., Font i Furnols M. (2010). Carcass and meat quality characteristics of immunocastrated male, surgically castrated male, entire male and female pigs. Meat Sci..

[26] Čandek-Potokar M., Škrlep M., Zamaratskaia G., Payan-Carreira R. (2017). Immunocastration as Alternative to Surgical Castration in Pigs. Theriogenology.

[27] Zamaratskaia G., Rydhmer L., Andersson H.K., Chen G., Lowagie S., Andersson K., Lundström K. (2008). Long-term effect of vaccination against gonadotropin-releasing hormone, using Improvac, on hormonal profile and behaviour of male pigs. Anim. Reprod. Sci..

[28] Rydhmer L., Lundström K., Andersson K. (2010). Immunocastration reduces aggressive and sexual behaviour in male pigs. Animal.

[29] Karaconji B., Lloyd B., Campbell N., Meaney D., Ahern T. (2015). Effect of an anti-gonadotropin-releasing factor vaccine on sexual and aggressive behaviour in male pigs during the finishing period under Australian field conditions. Aust. Vet. J..

[30] Cronin G.M., Dunshea F.R., Butler K.L., McCauley I., Barnett J.L., Hemsworth P.H. (2003). The effects of immuno- and surgical-castration on the behaviour and consequently growth of group-housed, male finisher pigs. Appl. Anim. Behav. Sci..

[31] Batorek N., Čandek-Potokar M., Bonneau M., Van Milgen J. (2012). Meta-analysis of the effect of immunocastration on production performance, reproductive organs and boar taint compounds in pigs. Animal.

[32] Claus R., Lacorn M., Danowski K., Pearce M.C., Bauer A. (2007). Short-term endocrine and metabolic reactions before and after second immunization against GnRH in boars. Vaccine.

[33] Batorek N., Škrlep M., Prunier A., Louveau I., Noblet J., Bonneau M., Čandek-Potokar M. (2012). Effect of feed restriction on hormones, performance, carcass traits, and meat quality in immunocastrated pigs. J. Anim. Sci..

[34] Metz C., Claus R. (2003). Active immunization of boars against GnRH does not affect growth hormone but lowers IGF-I in plasma. Livest. Prod. Sci..

[35] Batorek N., Noblet J., Dubois S., Bonneau M., Čandek-Potokar M., Labussiere E., Oltjen J.W., Kebreab E., Lapierre H. (2013). Effect of immunocastration in combination with addition of fat to diet on quantitative oxidation of nutrients and fat retention in male pigs. Proceedings of the Energy and Protein Metabolism and Nutrition in Sustainable Animal Production: 4th International Symposium on Energy and Protein Metabolism and Nutrition, Sacramento, CA, USA, 9–12 September 2013.

[36] Werner D., Baldinger L., Bussemas R., Büttner S., Weißmann F., Ciulu M., Mörlein J., Mörlein D. (2021). Early Immunocastration of Pigs: From Farming to Meat Quality. Animals.

[37] Andersson K., Brunius C., Zamaratskaia G., Lundström K. (2012). Early vaccination with Improvac^®^: Effects on performance and behaviour of male pigs. Animal.

[38] Lealiifano A.K., Pluske J.R., Nicholls R.R., Dunshea F.R., Campbell R.G., Hennessy D.P., Miller D.W., Hansen C.F., Mullan B.P. (2011). Reducing the length of time between slaughter and the secondary gonadotropin-releasing factor immunization improves growth performance and clears boar taint compounds in male finishing pigs. J. Anim. Sci..

[39] Škrlep M., Čandek-Potokar M., Batorek N., Šegula B., Prevolnik M., Pugliese C., Bonneau M.B. (2012). Length of the interval between immunocastration and slaughter in relation to boar taint and carcass traits. Acta Agric. Slov. Supl..

[40] Turkstra J.A., Zengt X.Y., van Diepent J.T., Jongbloed A.W., Oonk H.B., van de Wielt D.F., Meloen R.H. (2002). Performance of male pigs immunized against GnRH is related to the time of onset of biological response. J. Anim. Sci..

[41] Batorek-Lukač N., Čandek-Potokar M., Škrlep M., Kubale V., Labussière E. (2021). Effect of Changes in Dietary Net Energy Concentration on Growth Performance, Fat Deposition, Skatole Production, and Intestinal Morphology in Immunocastrated Male Pigs. Front. Vet. Sci..

[42] Trefan L., Doeschl-Wilson A., Rooke J.A., Terlouw C., Bünger L. (2013). Meta-analysis of effects of gender in combination with carcass weight and breed on pork quality. J. Anim. Sci..

[43] Pinna A., Schivazappa C., Virgili R., Parolari G. (2015). Effect of vaccination against gonadotropin-releasing hormone (GnRH) in heavy male pigs for Italian typical dry-cured ham production. Meat Sci..

[44] Font I.F.M., González J., Gispert M., Oliver M.A., Hortós M., Pérez J., Suárez P., Guerrero L. (2009). Sensory characterization of meat from pigs vaccinated against gonadotropin releasing factor compared to meat from surgically castrated, entire male and female pigs. Meat Sci..

[45] Font I.F.M., Gispert M., Soler J., Diaz M., Garcia-Regueiro J.A., Diaz I., Pearce M.C. (2012). Effect of vaccination against gonadotrophin-releasing factor on growth performance, carcass, meat and fat quality of male Duroc pigs for dry-cured ham production. Meat Sci..

[46] Škrlep M., Čandek-Potokar M., Lukač N.B., Povše M.P., Pugliese C., Labussière E., Flores M. (2016). Comparison of entire male and immunocastrated pigs for dry-cured ham production under two salting regimes. Meat Sci..

[47] Padmanabhan V., Cardoso R.C. (2020). Neuroendocrine, autocrine, and paracrine control of follicle-stimulating hormone secretion. Mol. Cell. Endocrinol..

[48] Pierce J.G., Parsons T.F. (1981). Glycoprotein hormones: Structure and function. Annu. Rev. Biochem..

[49] Rose M.P., Gaines Das R.E., Balen A.H. (2000). Definition and measurement of follicle stimulating hormone. Endocr. Rev..

[50] Andersen C.Y., Westergaard L.G., van Wely M. (2004). FSH isoform composition of commercial gonadotrophin preparations: A neglected aspect?. Reprod. Biomed. Online.

[51] Li C.H., Simpson M.E., Evans H.M. (1949). Isolation of Pituitary Follicle-Stimulating Hormone (FSH). Science.

[52] Gemzell C.A., Diczfalusy E., Tillinger G. (1958). Clinical effect of human pituitary follicle-stimulating hormone (FSH). J. Clin. Endocrinol. Metab..

[53] Kliesch S., Behre H.M., Nieschlag E. (1995). Recombinant human follicle-stimulating hormone and human chorionic gonadotropin for induction of spermatogenesis in a hypogonadotropic male. Fertil. Steril..

[54] Arce J.C., Andersen A.N., Fernández-Sánchez M., Visnova H., Bosch E., García-Velasco J.A., Barri P., de Sutter P., Klein B.M., Fauser B.C. (2014). Ovarian response to recombinant human follicle-stimulating hormone: A randomized, antimüllerian hormone-stratified, dose-response trial in women undergoing in vitro fertilization/intracytoplasmic sperm injection. Fertil. Steril..

[55] Kumar T.R., Wang Y., Lu N., Matzuk M.M. (1997). Follicle stimulating hormone is required for ovarian follicle maturation but not male fertility. Nat. Genet..

[56] Zaidi M., Yuen T., Kim S.M. (2023). Pituitary crosstalk with bone, adipose tissue and brain. Nat. Rev. Endocrinol..

[57] Liu X.M., Chan H.C., Ding G.L., Cai J., Song Y., Wang T.T., Zhang D., Chen H., Yu M.K., Wu Y.T. (2015). FSH regulates fat accumulation and redistribution in aging through the Gαi/Ca^2+^/CREB pathway. Aging Cell.

[58] Quinn M.A., Xu X., Ronfani M., Cidlowski J.A. (2018). Estrogen Deficiency Promotes Hepatic Steatosis via a Glucocorticoid Receptor-Dependent Mechanism in Mice. Cell Rep..

[59] Cui H., Zhao G., Liu R., Zheng M., Chen J., Wen J. (2012). FSH stimulates lipid biosynthesis in chicken adipose tissue by upregulating the expression of its receptor FSHR. J. Lipid Res..

[60] Kobayashi T., Pakarinen P., Torgersen J., Huhtaniemi I., Andersen Ø. (2008). The gonadotropin receptors FSH-R and LH-R of Atlantic halibut (*Hippoglossus hippoglossus*)—2. Differential follicle expression and asynchronous oogenesis. Gen. Comp. Endocrinol..

[61] Ubuka T., Bentley G.E., Tsutsui K. (2013). Neuroendocrine regulation of gonadotropin secretion in seasonally breeding birds. Front. Neurosci..

[62] Kumar R.S., Ijiri S., Kight K., Swanson P., Dittman A., Alok D., Zohar Y., Trant J.M. (2000). Cloning and functional expression of a thyrotropin receptor from the gonads of a vertebrate (bony fish): Potential thyroid-independent role for thyrotropin in reproduction. Mol. Cell. Endocrinol..

[63] Luo L., Liu M. (2016). Adipose tissue in control of metabolism. J. Endocrinol..

[64] Harvey I., Boudreau A., Stephens J.M. (2020). Adipose tissue in health and disease. Open Biol..

[65] Min H.K., Kapoor A., Fuchs M., Mirshahi F., Zhou H., Maher J., Kellum J., Warnick R., Contos M.J., Sanyal A.J. (2012). Increased hepatic synthesis and dysregulation of cholesterol metabolism is associated with the severity of nonalcoholic fatty liver disease. Cell Metab..

[66] Tian L., Song Y., Xing M., Zhang W., Ning G., Li X., Yu C., Qin C., Liu J., Tian X. (2010). A novel role for thyroid-stimulating hormone: Up-regulation of hepatic 3-hydroxy-3-methyl-glutaryl-coenzyme A reductase expression through the cyclic adenosine monophosphate/protein kinase A/cyclic adenosine monophosphate-responsive element binding protein pathway. Hepatology.

[67] Pertusa M., Morenilla-Palao C., Carteron C., Viana F., Cabedo H. (2007). Transcriptional control of cholesterol biosynthesis in Schwann cells by axonal neuregulin 1. J. Biol. Chem..

[68] Horton J.D., Goldstein J.L., Brown M.S. (2002). SREBPs: Activators of the complete program of cholesterol and fatty acid synthesis in the liver. J. Clin. Investig..

[69] Guo Y., Zhao M., Bo T., Ma S., Yuan Z., Chen W., He Z., Hou X., Liu J., Zhang Z. (2019). Blocking FSH inhibits hepatic cholesterol biosynthesis and reduces serum cholesterol. Cell Res..

[70] Qiao S., Alasmi S., Wyatt A., Wartenberg P., Wang H., Candlish M., Das D., Aoki M., Grünewald R., Zhou Z. (2023). Intra-pituitary follicle-stimulating hormone signaling regulates hepatic lipid metabolism in mice. Nat. Commun..

[71] Rucci N. (2008). Molecular biology of bone remodelling. Clin. Cases Miner. Bone Metab..

[72] Xiao W., Wang Y., Pacios S., Li S., Graves D.T. (2016). Cellular and Molecular Aspects of Bone Remodeling. Front. Oral Biol..

[73] Sun L., Peng Y., Sharrow A.C., Iqbal J., Zhang Z., Papachristou D.J., Zaidi S., Zhu L.L., Yaroslavskiy B.B., Zhou H. (2006). FSH directly regulates bone mass. Cell.

[74] Liu S., Cheng Y., Xu W., Bian Z. (2010). Protective Effects of Follicle-stimulating Hormone Inhibitor on Alveolar Bone Loss Resulting from Experimental Periapical Lesions in Ovariectomized Rats. J. Endod..

[75] Zhu L.L., Blair H., Cao J., Yuen T., Latif R., Guo L., Tourkova I.L., Li J., Davies T.F., Sun L. (2012). Blocking antibody to the β-subunit of FSH prevents bone loss by inhibiting bone resorption and stimulating bone synthesis. Proc. Natl. Acad. Sci. USA.

[76] Cannon J.G., Kraj B., Sloan G. (2011). Follicle-stimulating hormone promotes RANK expression on human monocytes. Cytokine.

[77] Cannon J.G., Cortez-Cooper M., Meaders E., Stallings J., Haddow S., Kraj B., Sloan G., Mulloy A. (2010). Follicle-stimulating hormone, interleukin-1, and bone density in adult women. Am. J. Physiol.-Regul. Integr. Comp. Physiol..

[78] Iqbal J., Sun L., Kumar T.R., Blair H.C., Zaidi M. (2006). Follicle-stimulating hormone stimulates TNF production from immune cells to enhance osteoblast and osteoclast formation. Proc. Natl. Acad. Sci. USA.

[79] Wu Y., Torchia J., Yao W., Lane N.E., Lanier L.L., Nakamura M.C., Humphrey M.B. (2007). Bone microenvironment specific roles of ITAM adapter signaling during bone remodeling induced by acute estrogen-deficiency. PLoS ONE.

[80] Chin K.Y. (2018). The Relationship between Follicle-stimulating Hormone and Bone Health: Alternative Explanation for Bone Loss beyond Oestrogen?. Int. J. Med. Sci..

[81] Carnac G., Vernus B., Bonnieu A. (2007). Myostatin in the pathophysiology of skeletal muscle. Curr. Genom..

[82] Ongaro L., Zhou X., Wang Y., Schultz H., Zhou Z., Buddle E.R.S., Brûlé E., Lin Y.F., Schang G., Hagg A. (2025). Muscle-derived myostatin is a major endocrine driver of follicle-stimulating hormone synthesis. Science.

[83] Molenaar G.J., Lugard-Kok C., Meloen R.H., Oonk R.B., de Koning J., Wensing C.J. (1993). Lesions in the hypothalamus after active immunisation against GnRH in the pig. J. Neuroimmunol..

[84] Han X., Meng F., Cao X., Du X., Bu G., Kong F., Huang A., Zeng X. (2021). FSH promotes fat accumulation by activating PPARγ signaling in surgically castrated, but not immunocastrated, male pigs. Theriogenology.

[85] Huang T., Yang M., Dong K., Xu M., Liu J., Chen Z., Zhu S., Chen W., Yin J., Jin K. (2020). A transcriptional landscape of 28 porcine tissues obtained by super deepSAGE sequencing. BMC Genom..

[86] Dunshea F.R., Allison J.R.D., Bertram M., Boler D.D., Brossard L., Campbell R., Crane J.P., Hennessy D.P., Huber L., de Lange C. (2013). The effect of immunization against GnRF on nutrient requirements of male pigs: A review. Animal.

[87] Bauer A., Lacorn M., Danowski K., Claus R. (2008). Effects of immunization against GnRH on gonadotropins, the GH-IGF-I-axis and metabolic parameters in barrows. Animal.

[88] Cui H., Zhao G., Wen J., Tong W. (2018). Follicle-stimulating hormone promotes the transformation of cholesterol to estrogen in mouse adipose tissue. Biochem. Biophys. Res. Commun..

[89] Yao Y.C., Cai Z.W., Zhao C.J., Wu K.L., Wu C.X., Han W.P., Xu N.Y. (2011). Influence of castration-induced sex hormone deficiency on serum lipid levels and the genes expression in male pigs. Horm. Metab. Res..

[90] Grasso P., Rozhavskaya M., Reichert L.E. (1998). In Vivo Effects of Human Follicle-Stimulating Hormone-Related Synthetic Peptide hFSH-,β-(81–95) and Its Subdomain hFSH-β-(90–95) on the Mouse Estrous Cycle. Biol. Reprod..

[91] Han X., Guan Z., Xu M., Zhang Y., Yao H., Meng F., Zhuo Y., Yu G., Cao X., Du X. (2020). A novel follicle-stimulating hormone vaccine for controlling fat accumulation. Theriogenology.

[92] Ji Y., Liu P., Yuen T., Haider S., He J., Romero R., Chen H., Bloch M., Kim S.M., Lizneva D. (2018). Epitope-specific monoclonal antibodies to FSHβ increase bone mass. Proc. Natl. Acad. Sci. USA.

[93] Di Pasquale J., Nannoni E., Sardi L., Rubini G., Salvatore R., Bartoli L., Adinolfi F., Martelli G. (2019). Towards the Abandonment of Surgical Castration in Pigs: How is Immunocastration Perceived by Italian Consumers?. Animals.

